# Stacked Autoencoders for Outlier Detection in Over-the-Horizon Radar Signals

**DOI:** 10.1155/2017/5891417

**Published:** 2017-10-23

**Authors:** Eftychios Protopapadakis, Athanasios Voulodimos, Anastasios Doulamis, Nikolaos Doulamis, Dimitrios Dres, Matthaios Bimpas

**Affiliations:** ^1^National Technical University of Athens, 15780 Athens, Greece; ^2^Department of Informatics, Technological Educational Institute of Athens, 12243 Athens, Greece; ^3^Telesto Technologies, 15561 Cholargos, Greece

## Abstract

Detection of outliers in radar signals is a considerable challenge in maritime surveillance applications. High-Frequency Surface-Wave (HFSW) radars have attracted significant interest as potential tools for long-range target identification and outlier detection at over-the-horizon (OTH) distances. However, a number of disadvantages, such as their low spatial resolution and presence of clutter, have a negative impact on their accuracy. In this paper, we explore the applicability of deep learning techniques for detecting deviations from the norm in behavioral patterns of vessels (outliers) as they are tracked from an OTH radar. The proposed methodology exploits the nonlinear mapping capabilities of deep stacked autoencoders in combination with density-based clustering. A comparative experimental evaluation of the approach shows promising results in terms of the proposed methodology's performance.

## 1. Introduction

Detection of targets and outliers in radar signals is a research issue that has gained significant attention in the academic and industrial research community, mainly because of the important associated impact of relevant applications in surveying of large areas. High-Frequency Surface-Wave (HFSW) radars are a category of radars that operate at the frequency band 3–30 MHz and, in contrast with other radars, use ground wave or sky wave propagation and ionospheric reflections of the electromagnetic waves for target detection, which allows for achieving longer ranges, where microwave radars cannot perform [1], but to the detriment of the attained accuracy. For many years, HFSW radars, or over-the-horizon (OTH) radars, as they are commonly known, have been used to remotely measure oceanographic parameters, providing information about surface currents, wave spectra, wind direction and intensity, and so on [2]. Their extraordinary range (up to 200 nautical miles) combined with their continuous mode of operation make for an ideal candidate tool for long-range oceanic surveillance. However, many associated weaknesses, for example, low spatial resolution, high nonlinearity, and important presence of clutter, negatively impact their performance as early-warning tools for detection, tracking, and identification of vessels.

The promising capabilities of OTH radars have attracted significant interest from the research community and have already resulted in various approaches (e.g., [3, 4]). Nevertheless, related research issues continue to present significant challenges, which can be attributed to few reasons, briefly described below:

- Different targets may present similar dielectric and frequency properties thus making it hard to make a clear distinction among them.
- Given multipath propagation effects of rough surfaces, scattering from some objects tends to overwhelm the weak backscattering of targets.
- Due to the changes in atmosphere and ground conditions, noise is added which can confuse the analysis of a radar signal.
- Ocean and ionospheric clutter generate noise especially for HFSW radars.

On a different note, the surge of deep learning and the great results it has produced in other signal analysis domains, such as computer vision, speech recognition, and natural language processing, create certain expectations regarding its potential efficacy in radar signal analysis applications. Deep learning allows computational models of multiple processing layers to learn and represent data with multiple levels of abstraction mimicking how the brain perceives and processes multimodal information, thereby implicitly capturing intricate structures of large‐scale data. Complex abstractions are learnt at a given level based on relatively simpler abstractions formulated in the preceding layer in the hierarchy.

The goal of this paper is to present a framework for detecting deviations from the norm in behavioral patterns of vessels (henceforth called* outliers*), as they are tracked from an OTH radar. The proposed methodology exploits the nonlinear mapping capabilities of deep stacked autoencoders (SAs) [5] in combination with density-based clustering. Stacked autoencoders are used in an unsupervised way to map the track history of any vessel into a compact and informative feature vector. Then, at any moment all tracked ships are projected into a new feature space and clustered using density-based algorithms, such as OPTICS [6]. The outcome of the clustering stage then indicates possible outliers.

The remainder of this paper is structured as follows: Section 2 presents an overview of the related work. In Section 3 we describe in detail the proposed methodology for outlier detection in OTH radar signals, which is followed by the experimental evaluation of the methodology in Section 4. Finally, Section 5 concludes the paper.

## 2. Related Work

In the literature, several signal processing and machine learning methods have been investigated and proposed to acquire more reliable data with lower noise and extract semantic information from radar signals. Kouemou and Opitz [7] introduced a wavelet-based feature analysis combined with Hidden Markov Models (HMM) to classify real radar signals into predefined categories. Spectral analysis [8] is used by Garbanzo-Salas and Hocking [9] for detecting small objects from harmonic pulse radar data. The use of online bootstrapping machine learning tools to improve target detection rate of radar signals is also one major research area [10]. Radar data can be analyzed using the concepts of transfer learning since often we have only a small number of labelled data available while the majority of signals captured are unlabelled (nonannotated) [11]. Other works focus on modeling of ionospheric disturbances on spaceborne interferometric synthetic aperture radar (SAR) via Echo-State Networks [12, 13] or ensemble classifiers [14].

Denoising techniques for radar signals include low level processing such as the median filter or other nonlinear convolution schemes [15]. Other approaches spatially or temporally decompose radar signals by wavelet transforms [16, 17]. This way, we can find patterns distributed on space and time domain to improve targets detection efficiency. These methodologies can be extended to the analysis of synthetic aperture radar (SAR) images [11], or by incorporating sparsity-based signal analysis [18]. A neural network based scheme for detecting salient objects in SAR images is recently presented [19]. The goal is to identify changes in SAR content. A similar approach for detecting changes using nonlinear stacked restricted Boltzmann machines is given in the work of Liu et al. [20], while multilayered feature learning to improve detection accuracy of SAR images is described by Xie et al. [21]. Furthermore, low-power HF surface-wave (HFSW) radars have demonstrated being a cost-effective long-range early-warning sensor for ship detection and tracking [22, 23]. A detailed description of various ways in which HFSW radar technology can be used for maritime surveillance is provided by Braca et al. [24].

Regarding deep versus “shallow” learning schemes, traditional machine learning techniques exploit shallow architectures; that is, they use a single layer for data/feature transformation, even in a highly nonlinear space. Shallowness refers here to the simplicity of these architectures that use only one (or few) layer(s) of processing, responsible for transforming the raw input signals or features into the problem-specific feature space. Instead, in a deep learning paradigm, the architectures are composed of many (deep) nonlinear processing stages [25]. Deep learning has been extensively applied in many fields, such as computer vision [26] (e.g., behavior recognition [27] and human tracking [28]) and speech recognition [29]. However, its applicability in radar signal processing had not being investigated until very recently [30]. Even so, most of the proposed works pertain to object detection in SAR image data [31], essentially resembling visual analysis approaches.

## 3. The Proposed Methodology

The proposed methodology exploits the nonlinear mapping abilities of stacked autoencoders (SAs) [5] in combination with density-based clustering, to identify irregular occurrences, using over-the-horizon radar data. Such an approach is based on two main assumptions:

1. The history of a naval vessel, in terms of speed, position, course, signal frequency, or other related data, provided by a ground radar, suffices to extract meaningful features.
2. Unexpected deviation from the norm is observed for a few ships, denoted henceforth as outliers.

**Figure 1:**
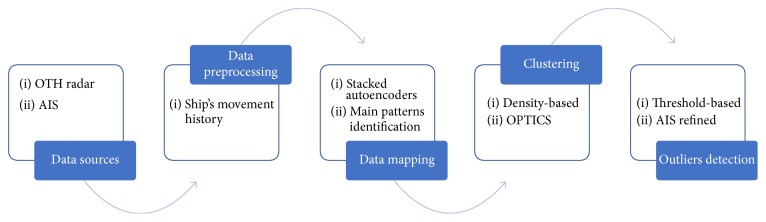
Proposed approach flowchart.

The approach is relatively straightforward: Given a set of OTH data entries, SAs are used in an unsupervised way to map the track history of any vessel into a compact and informative feature vector. Then, at any moment all tracked ships are projected into a new feature space and clustered using OPTICS [6], a widely used density-based algorithm. The clustering outcome informs about possible outliers. In the following subsections, the different stages of the methodology are presented, after a brief description of the data involved.  Figure 1 provides a high-level view of the proposed approach.

### 3.1. OTH and AIS Data

Heterogeneous data, such as automatic identification system (AIS) data, high-frequency surface wave (HFSW) radar data, and synthetic aperture radar (SAR) data, have been exploited in research for maritime surveillance purposes [32]. In our case, two sources of information were fused to support the outlier detection process: OTH radar and AIS data.

The OTH radar data used for the setting and evaluation of the presented work was acquired by the HFSW STRADIVARIUS radar by Diginext [33]. OTH radar detection (plot) and tracking (track) data are the output of the OTH radar for a given period. The plot and track data provided include estimated position coordinates, velocity, course, Doppler frequency, global and local noise levels, azimuth, and other parameters, appropriately timestamped.

On a different note, AIS is an automatic tracking system used for collision avoidance on ships and by vessel traffic services. AIS information supplements marine radar, which continues to be the primary method of collision avoidance for water transport. Vessels equipped with AIS transceivers can be tracked by AIS base stations located along coast lines. The International Maritime Organization's International Convention for the Safety of Life at Sea requires AIS to be present aboard international voyaging ships with gross tonnage of 300 or more and all passenger ships regardless of size [34]. AIS reports contain both dynamic information (e.g., latitude, longitude, course over ground, speed over ground, and time) and static information (e.g., vessel type and dimension information).

### 3.2. Density-Based Clustering as a Basis for Outlier Detection

Clustering refers to the task of identifying groups or clusters in a dataset. In density-based clustering, a cluster is a set of data objects spread in the data space over a contiguous region of high density of objects. Density-based clusters are separated from each other by contiguous regions of low density of objects. Data objects located in low-density regions are typically considered noise or outliers [35]. OPTICS algorithm [6], as one among various approaches for hierarchical density-based clustering, includes ordering points to identify the clustering structure. OPTICS is based on DBSCAN [36] and the work of Stuetzle [37].

OPTICS computes a Minimum Spanning Tree (MST) of the data, where edge weights represent pairwise distances. These distances are smoothed by a density estimator, called core distance. The core distance of a point **x**_*i*_ is the smallest threshold *r* such that **x**_*i*_ is still considered a core object by the DBSCAN algorithm; that is, **x**_*i*_ has at least *k* objects in its neighborhood within radius *r*. The resulting distance, which is used to construct the MST, is called reachability distance (RD). Taking *k* as input parameter for smoothing the density estimation, the reachability distance of point **x**_*i*_ is defined relative to a reference object **y** as the minimum of the core distance of **y** and the actual distance between **x**_*i*_ and **y**. The outcome of the algorithm can provide us information about the clustering of the objects (see Section 3.4).

### 3.3. Using Stacked Autoencoders for Data Representation

Density-based algorithms, traditionally, use the Euclidian distance metric [38]. Such distance metrics are prone to high dimensionality related problems. If we have a feature space of many dimensions, that is, the tracked course of a ship, clustering performance decreases.

Let **n** and **m** be points drawn from a* d*-dimensional Gaussian distribution, so that **n** ~ *N*(*μ*_1_, *σ*_1_^2^ · **I**) and **m** ~ *N*(*μ*_2_, *σ*_2_^2^ · **I**). Then their expected distance satisfies [39]

(1) En−m2=E∑i=1dni−mi2=∑i=1dVarni−mi+Eni−mi2=d·σ12+σ22+μ1−μ22.

Thus, the term *d* · (*σ*_1_^2^ + *σ*_2_^2^), where *d* is a scalar denoting the dimensions of the Gaussian distribution, overshadows the informative term ‖*μ*_1_ − *μ*_2_‖^2^. At this point, the need of robust low-dimension features becomes apparent. In such cases the use of autoencoders is advised [5].

An autoencoder is a neural network that is trained to attempt to copy its input to its output. Internally, it has a hidden layer *h* that describes a code used to represent the input. The network may be viewed as consisting of two parts: an encoder function **h** = *f*(**x**) and a decoder that produces a reconstruction **r** = *g*(**h**). Autoencoders are designed to be unable to learn to copy perfectly, since they are trained such that *g*(*f*(**x**)) ≈ **x** instead of (*f*(**x**)) = **x**. The model often learns useful properties of the data, because it is forced to prioritize which aspects of the input should be copied.

Usually, training the autoencoder to perform the input copying task will result in *h* taking on useful properties, constraining *h* to have smaller dimension than **x**. An autoencoder whose code dimension is less than the input dimension is called undercomplete. Learning an undercomplete representation forces the autoencoder to capture the most salient features of the training data

The learning process is described simply as minimizing a loss function, for example, *L*(**x**, *g*(*f*(**x**))), where *L* is a loss function penalizing *g*(*f*(**x**)) or being dissimilar from **x**, such as the mean squared error. When the decoder is linear and *L* is the mean squared error, an undercomplete autoencoder learns to span the same subspace as PCA. In this case, an autoencoder trained to perform the copying task has learnt the principal subspace of the training data as a side effect

A sparse autoencoder is simply an autoencoder whose training criterion involves a sparsity penalty *Ω*(**h**) on the code layer **h**, in addition to the reconstruction error, that is, *L*(**x**, *g*(*f*(**x**))) + *Ω*(**h**). Sparse autoencoders are typically used to learn features for another task such as classification. An autoencoder that has been regularized to be sparse must respond to unique statistical features of the dataset it has been trained on, rather than simply acting as an identity function.

The core idea of our work lies in using stacked autoencoders to capture a representation of the main patterns present in the data. By doing so, any outlier in data samples will not be explained well using that representation. In other words, outliers will have significant variations from the rest of the data.

### 3.4. Identifying Outliers

The outlier detection is a combinatory threshold-based approach built on the interquartile range rule, as in [40], OPTICS output (see Section 3.2), and AIS/OTH matched data (see Section 3.5).

OPTICS outputs (i.e., reachability distances of the ordered ships) are treated as a continuous signal, over which we identify the peaks. Peaks correspond to significant changes between the closest compared vehicles. As such, anything that varies from the norm has a peak, allowing the easy identification of a possible outlier. Then, we calculate a threshold value ths^(*t*)^ defined as ths^(*t*)^ = (1/*m*)∑_*i*_^*m*^**RD**_*o*_(*i*), *m* = ⌈0.1 · *n*_*t*_⌉, where *n*_*t*_ denotes the number of ships at a time *t* and **RD**_*o*_ is the reachability distances vector, in a descending order.

**Figure 2:**
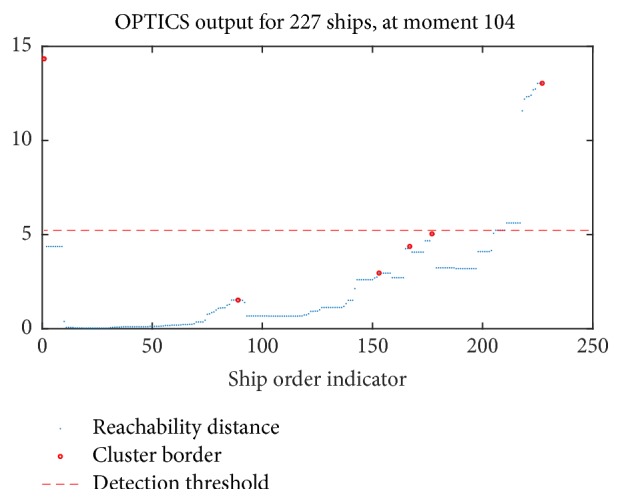
(Best viewed in color) illustration of an instance of the outlier detection mechanism at a specific time moment.

In case that an outlier provides AIS data, the detection regarding that ship is ignored. At first, for a specific time instance, ships are ordered in a density-reachable way (Figure 2). Points close to each other should belong to the same cluster, unless there is a significant change in RD value. Then, the outlier RD value threshold is defined over 10% of highest RDs.

### 3.5. Matching OTH Data to AIS

As explained in Section 3.1, AIS data contain, among others, ships' trajectory points. These coordinates are compared to the radar ones, to identify the similarity among the trajectories. Let us denote as **T**_*R*_^(*v*_*i*_)^ = [*t*_1_,…, *t*_*p*_] the available discrete time instances, created from the ground radar for ship *v*_*i*_, *i* = 1,…, *n*. The equivalent case for AIS data is **T**_AIS_^(*𝓋*_*j*_)^ = [*t*_1_,…, *t*_*q*_] for any ship *𝓋*_*j*_, *j* = 1,…, *l*, that provides AIS data.

**Figure 3:**
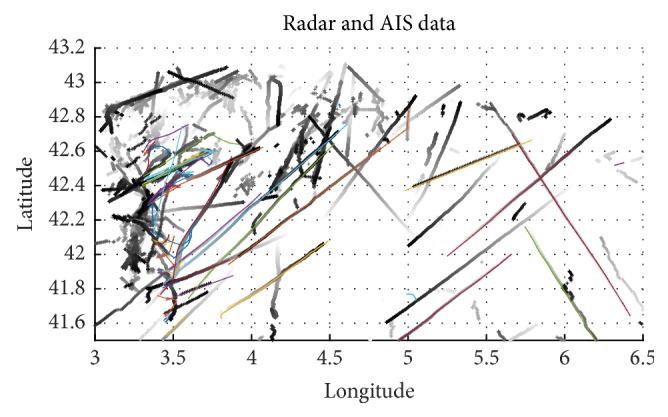
(Best viewed in color) an illustration of the investigated ship trajectories. Ground radar trajectories are plotted in grayscale. The fading colors correspond to past times.

**Figure 4:**
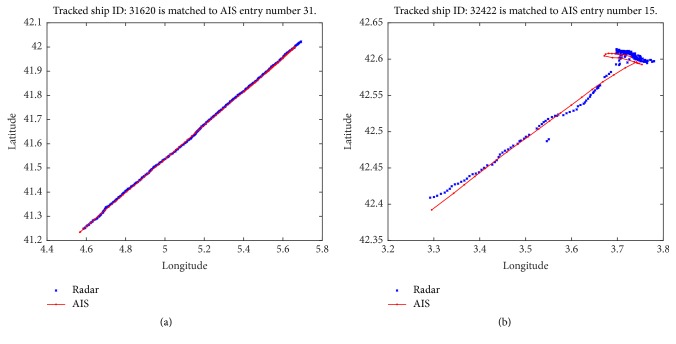
(Best viewed in color) illustration of matched trajectories between ground radar and AIS data (a) and matched trajectories despite the noise, due to minor course deviations (b).

Figure 3 illustrates the available trajectories over a specified area for both radar and AIS data. At this point, we should note that trajectories are calculated for various time intervals, which do not, usually, coincide among the two systems. Typically, for the same ship *p* > *q*, in a ratio of four radar time instances to one AIS time instance. Also, note that *l* < *n*, so that a 1-to-1 match among radar and AIS tracked ships is not feasible. Therefore, we should consider both the temporal and the spatial information, to find the matches. The algorithm (presented in pseudocode in Algorithm 1) performs the vessel matching (Figure 4), given OTH and AIS information recorded at the same time (for further details about used data see Section 4.2).

The matching process is based on a voting mechanism. For each of the radar tracked ships *v*_*i*_, at a time instance *t*_*w*_, *w* = 1,…, *p*, we calculate the *k* closest ships *𝓋*_*i*_, according to their AIS position at the specific time. In order to identify the corresponding (closest) AIS time instance of ship *𝓋*_*i*_ to radar entry *v*_*i*_, at a time *t*_*w*_^*R*^, we calculate the time difference **T**_diff_^*𝓋*_*i*_  ^ = [*t*_1_^AIS^ − *t*_*w*_^*R*^,…, *t*_*q*_^AIS^ − *t*_*w*_^*R*^]; then the corresponding time instance is given as *c*_*t*_ = arg⁡min_**T**_diff_(*m*)≥0_⁡{**T**_diff_^*𝓋*_*i*_  ^(1),…, **T**_diff_^*𝓋*_*i*_  ^(*q*)}. In case that *c*_*t*_ = *∅*, AIS entry *𝓋*_*j*_ is not matched to *v*_*i*_ at time *t*_*w*_. Then, once we have a set of matched ship instances *M*_*v*_*i*__^*t*_*j*_^ = {*𝓋*_*r*_}_*r*=1_^*R*^, *R* < *q*, we find *k* closest entries to *v*_*i*_ according to their position (i.e., longitude, latitude), so that *M*_*v*_*i*__^*t*_*w*_^ = {*𝓋*_*r*_}_*r*=1_^*k*^, *k* ≪ *q*.

## 4. Experimental Results

In the following subsections, we describe the dataset utilized for the experiments, the performance evaluation metrics employed, and the system setup details, before presenting the experimental evaluation of the proposed framework.

### 4.1. Computational Complexity

Data preprocessing creates a set of *𝓀* OTH data related entries, for a predefined set of past moments, for each one of the *n*_*t*_ tracked ships, at a moment *t*. Since both *𝓀* and *𝓂* are constants defined by the user, the required runtime is *O*(*n*). The mapping process of a trained SA is *O*(1) per datum, since SAs are neural networks with a defined number of neurons. OPTICS processes each point once and performs one *ϵ*-neighborhood query during this processing. Given a spatial index that grants a neighborhood query in *O*(log⁡(*n*)) runtime, an overall runtime of *O*(*n* · log⁡(*n*)) is obtained. The matching process between AIS and OTH entries requires an overall runtime of *O*(*n* · *m*), *m*_*t*_ ≪ *n*_*t*_, since we compare each of the *n*_*t*_ OTH tracked ships to each of the *m*_*t*_ ships equipped with AIS.  Table 1 displays the computational complexity of the different processing steps.

### 4.2. Utilized Dataset

The utilized dataset pertains to approximately 6 hours of data captured from the Mediterranean coast of France by Diginext in July 2016 in the context of the RANGER EU Horizon 2020 project. AIS data for the same period were also obtained for use as ground truth.

A total of 556 ship entries were in this 6-hour dataset. The following data provided entries are used:

1. Longitude and latitude: position values provided in degrees. The typical range is [−180,180] and [−90,90], respectively
2. Course and speed: course is calculated in degrees, typically in the range [−180,180], and speed in m/s
3. Doppler frequency: it is calculated in Hz, typically in the range [−0.5, 0.5]
4. Raw Rx azimuth: azimuth angle from the Rx site in the raw spatial grid (equivalent to the reception beam), typically in the range [110,230]
5. Local noise: noise level in the surrounding of the plot. It is calculated in dBm, in the range [−120, −40]
6. Global noise: background noise level of all range-Doppler map. It is calculated in dBm, in the range [−120, −80].

### 4.3. Performance Metrics

Formally, a cluster analysis can be described as the partitioning a number of *N* classification objects in *K* groups or clusters {*C*_*k*_}, *k* = 1,…, *K*. Given *N* objects **X** = {**x**_1_, …, **x**_*N*_}, where **x**_*ij*_ denotes the *j*th element of **x**_*i*_. The grouping of all objects **x**_*i*_, *i* = 1, …, *N*, in *K* clusters can be defined as follows:

(2) $$\begin{array}{c} w_{ki}=\left\{\begin{array}{cc} 1, & \text{iff }x_{i}\in C_{k}\\ 0, & \text{otherwise.} \end{array}\right. \end{array}$$

The above formulation ensures that the association of each object to a cluster is unique. A unique association is a valid case for both hierarchical and partitioning cluster analysis. Given matrix **W**, various internal quality indices have been calculated, to determine an optimal clustering.

#### 4.3.1. Calinski–Harabasz Index

The Calinski–Harabasz index (CHI) [41] is defined according to the following equation:

(3) $$\begin{array}{c} CHI\left(\;k\;\right)=\frac{T_{B}/\left(K-1\right)}{T_{W}/\left(N-K\right)}, \end{array}$$

where *T*_**B**_ is defined as

(4) $$\begin{array}{c} T_{B}=\overset{k}{\underset{k=1}{\sum }}\left|\;{\overset{-}{C}}_{k}\;\right|\left\|\;C_{k}-\overset{-}{x}\;\right\| \end{array}$$

and *T*_**W**_ is defined as

(5) $$\begin{array}{c} T_{W}=\overset{k}{\underset{k=1}{\sum }}\;\overset{N}{\underset{i=1}{\sum }}w_{ki}{\left\|\;x_{i}-{\overset{-}{C}}_{k}\;\right\|}^{2}. \end{array}$$

*T*_**W**_ starts at a comparably large value. With increasing number of clusters *k*, approaching the optimal clustering solution in *K*^*∗*^ groups, the value should significantly decrease due to increasing compactness of each cluster. As soon as the optimal solution is exceeded an increase in compactness and thereby a decrease in value might still occur. However, any decrease in value should be notably smaller.

Calculated for each possible cluster solution, the maximum CHI value indicates the best cluster partitioning of the data.

#### 4.3.2. Davies–Bouldin Index

The Davies–Bouldin index (DBI) [42] is an internal evaluation scheme, where the validation of how well the clustering has been done is made using quantities and features inherent to the dataset. DBI is defined as follows:

(6) $$\begin{array}{c} DB\left(\;k\;\right)=\frac{1}{K}\overset{K}{\underset{k=1}{\sum }}R_{k}, \end{array}$$

where *R*_*k*_ is defined as

(7) $$\begin{array}{c} R_{k}=\max\left(\;\frac{S_{k}+S_{j}}{d_{kj}}\;\right),\;j=1,\ldots ,K\;\;j\neq k. \end{array}$$

*d*_*kj*_ is a distance function, defined as $d_{kj}=\left\|{\overset{-}{x}}_{k}-{\overset{-}{x}}_{j}\right\|$, and *𝒮*_*k*_ is defined as

(8) $$\begin{array}{c} S_{k}=\frac{1}{\sum_{i=1}^{N}w_{ki}}\overset{N}{\underset{i=1}{\sum }}w_{ki}\left\|\;x_{i}-{\overset{-}{x}}_{k}\;\right\|. \end{array}$$

All the above equations assume that *k* ∈ [1, *K*].

For each cluster *C*_*k*_ an utmost similar cluster—regarding their intracluster error sum of squares—is searched, leading to *R*_*k*_. The index then defines the average over these values. In this case, the minimum index value corresponds to the best cluster solution.

#### 4.3.3. Silhouette

The silhouette value is a measure of how similar an object is to its own cluster (cohesion) compared to other clusters (separation). The silhouette ranges from −1 to 1, where a high value indicates that the object is well matched to its own cluster and poorly matched to neighboring clusters. If most objects have a high value, then the clustering configuration is appropriate. If many points have a low or negative value, then the clustering configuration may have too many or too few clusters.

For each datum **x**_*i*_, let *α*(**x**_*i*_) be the average dissimilarity (distance) of **x**_*i*_ with all other data within the same cluster *C*_*k*_. Let *b*(**x**_*i*_) be the lowest average dissimilarity of **x**_*i*_ to any other cluster *C*_*l*_, *l* ≠ *k*, of which **x**_*i*_ is not a member. We now define a silhouette as

(9) $$\begin{array}{c} s\left(\;x_{i}\;\right)=\frac{b\left(x_{i}\right)-\alpha \left(x_{i}\right)}{\max\left\{\alpha \left(x_{i}\right),b\left(x_{i}\right)\right\}}; \end{array}$$

thus, *s*(**x**_*i*_) ∈ [−1,1]. Values close to one indicate that the datum **x**_*i*_ is appropriately clustered at *C*_*k*_. The average silhouette value over all data, that is, $\overset{-}{s}=\left(1/n\right)\sum_{i=1}^{n}s\left(x_{i}\right)$, is another measurement for the quality of the generated clusters.

### 4.4. Experimental Setup

The first step should be the definition of the feature space on which radar data are mapped. As a starting point, we investigated the dimensional space provided by PCA, maintaining 99.1% of the original variation. The adopted stacked autoencoder approach consists of three layers or four layers, depending on the PCA outcome. The loss function was the well-known mean square error [43] with L2 and sparsity regularizers [44].

Ships track history is composed of 9 consecutive frames, each containing all data as described in Section 4.2. Data are normalized using min-max approach, prior to mapping or clustering approach. The system ignores ships with a narrow appearance span. Any ship that has no enough sufficient entries, that is, 3/4 of past moments tracks, is not taken under consideration.

### 4.5. Evaluation of Results

OPTICS algorithm outcomes depend on the selection of minimum cluster size. We have investigated the clustering outputs assuming at least 2, 5, 8, 11, 14, 17, 20, 23, and 26 members in each cluster. Clustering over SA mapped data performed better than using raw or PCA mapped data, for most of investigated cases.

**Figure 5:**
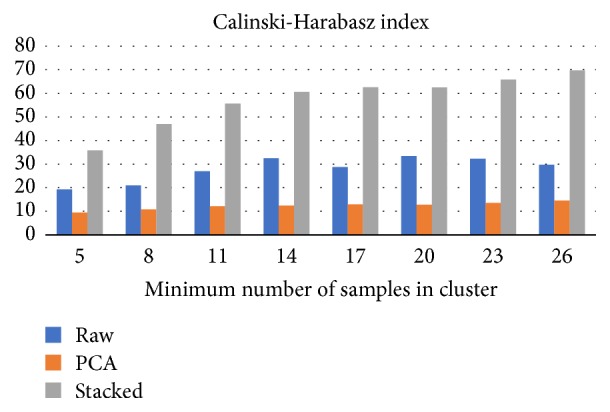
The impact of minimum cluster size (OPTICS input parameter) on Calinski–Harabasz index average score. Stacked autoencoders CHI scores are better in all the investigated cases, compared to PCA and raw data based clusters.

According to CHI (Figure 5), highest scores are achieved when using 26 ships per cluster. It is intriguing that cluster performance scores over raw data outperform PCA mapped data scores. There is an increasing trend on the CHI as the minimum cluster size increases. The trend is clearly illustrated for SAs, less for raw data, and slightly for PCA projected data.

**Figure 6:**
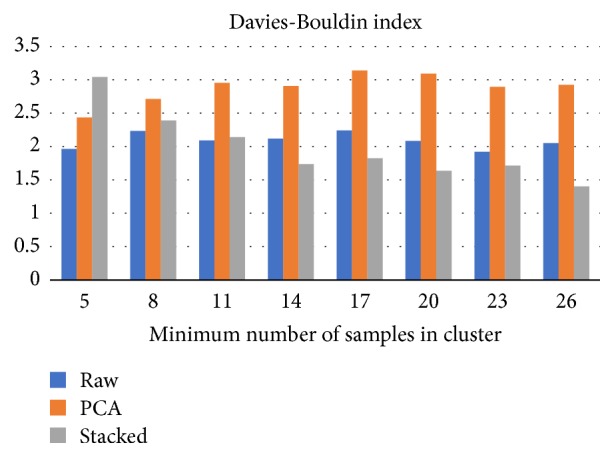
The impact of minimum cluster size (OPTICS input parameter) on Davies–Bouldin index average score. Stacked autoencoders CHI scores are better in six out of eight investigated cases, compared to PCA based clusters, and five out of eight cases compared to raw data.

The next step was the investigation of DBI scores for the same minimum cluster size setup (Figure 6). This time, the best scores are achieved using 14 or 20 as the cluster size. SA mapping provides better clustering scores in five out of seven investigated cases. Regardless of the mapping method, CHI scores, over SA mapped data, improve as the number of clusters rises, but not in a monotonic way.

**Figure 7:**
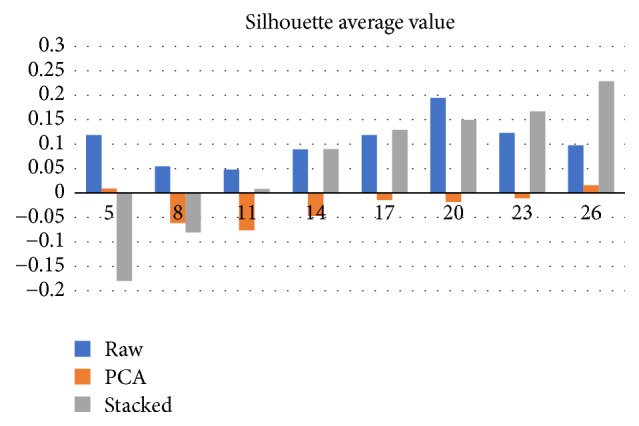
Impact of minimum cluster size (OPTICS input parameter) on silhouette average values. Stacked autoencoders silhouette scores are better in five out of eight investigated cases, compared to raw based clusters.

The last cluster performance metric was the average silhouette distance (Figure 7). Results suggest that accepting two ships as minimum cluster size is the best possible setup, for PCA mapped data. On the other hand, if we use SA for data mapping, the minimum cluster size should be set as 20.

Another significant performance metric is the average reachability distance itself. The smaller the reachability distance of a point is, the higher the density is around it. The core idea of the proposed approach is that only outliers should vary significantly from the norm, on the projected feature space. Thus, all the ships, minus the outliers, should have similar feature values, which results in reduced reachability distances.

**Figure 8:**
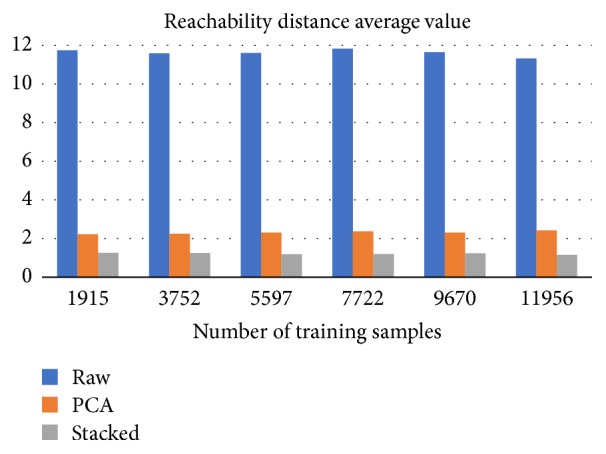
An illustration of how the number of training paradigms affects the average reachability distances (OPTICS outputs). Raw data average RD value exceeds 10, in each of the cases.

**Figure 9:**
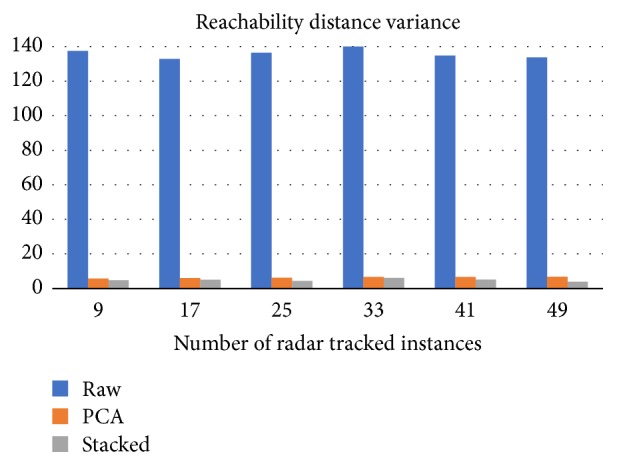
Illustration of the training period span effect on the variance in reachability distances. Raw data RD variance exceeds 40, in each case.

**Figure 10:**
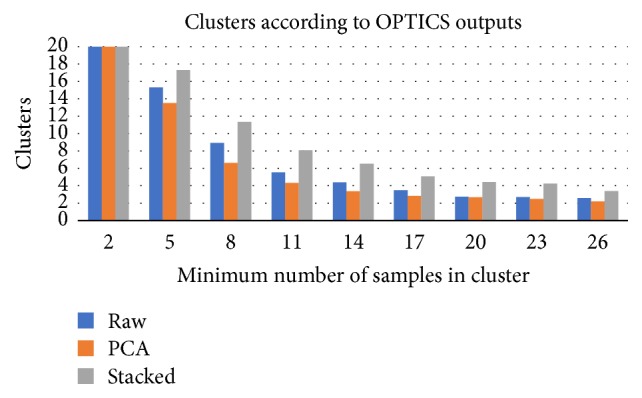
Average number of generated clusters given various mapping approaches. In all of the investigated cases (i.e., different minimum cluster size), SAs provide more clusters.

Providing more training data allows SA to adjust the mapping process to the norm. As illustrated in Figure 8 the average reachability distance tends to one, at a slow pace, while increasing the number of training samples. The variance of the RD is, also, reduced when using more time instances for training, as shown in Figure 9. Furthermore, SA mapping allows for the creation of more clusters compared to PCA or raw data clustering (Figure 10).

**Figure 11:**
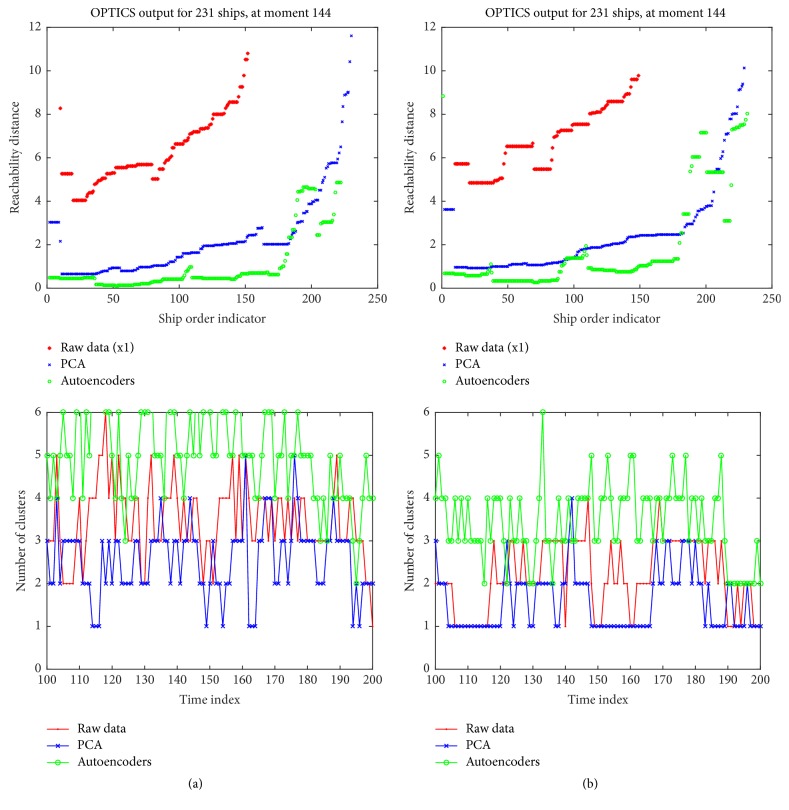
(Best viewed in color) comparison of OPTICS outputs over the same time instance, setting as minimum cluster size (a) 20 and (b) 26 ships. Stacked autoencoders result in more clusters than PCA or raw data that implies more peaks in the signal, which leads to more outliers' detection.

Regardless of the adopted feature mapping approach, OPTICS outputs are at least four times less in value, compared to calculated RDs using raw data (see Figures 11(a) and 11(b), top). Additionally, SAs result in more clusters, in most of the cases (see Figures 11(a) and 11(b), bottom). Increasing the number of minimum ships per cluster, close objects have almost identical reachability distances, resulting in almost linear subregions, within RD curve.

**Figure 12:**
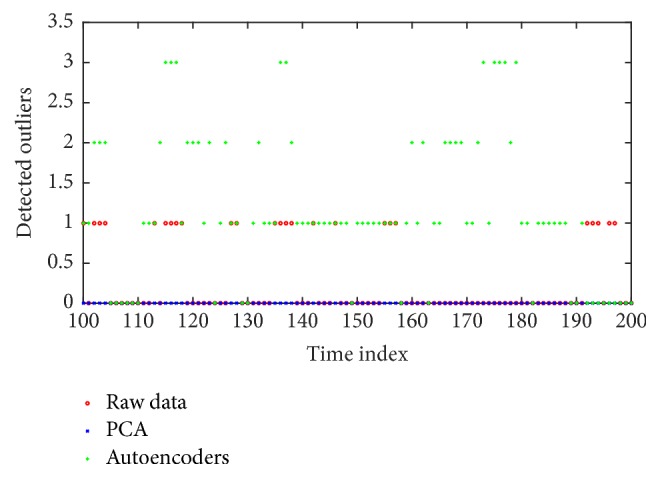
(Best viewed in color) illustration of the detected outliers through time. Using SAs' mapped data results in more outliers compared to the other approaches. Some of the selected outliers correspond to ships equipped with AIS transmitters.

The last step of the performance analysis provides empirical findings. In most of the cases, SAs mapped data results in detection of more outliers compared to the other approaches (Figure 12). The maximum number of detected outliers was three. PCA resulted in no detection at any time.

**Figure 13:**
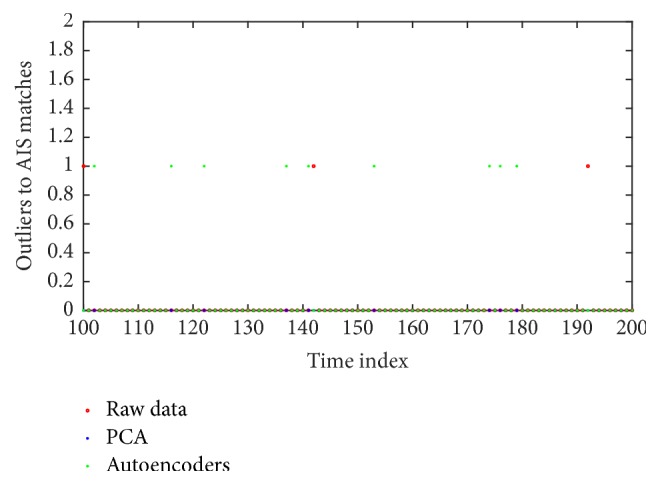
(Best viewed in color) illustration of the ships identified as possible outliers, while providing AIS data. Such cases are not considered as outliers.

There was the possibility of unwanted outlier identification. In particular, ships providing AIS data were considered, a few times, possible outliers.  Figure 13 illustrates the case. Typically, using SAs resulted in few possible outliers, which however were not accepted as valid detection, as explained in Section 3.4.

## 5. Conclusions

In our article, a novel approach that identifies unexpected behavior in ship plot and track patterns, as captured by an OTH radar, has been presented. The core idea is the unsupervised development of a mapping process, which can project the raw data in a compact, lower feature space. Outliers projected to the same space should have significantly different values. Stacked autoencoders and PCA were used for the mapping process and compared against the exploitation of raw data, for the identification of unusual ship behavior. Density-based clustering algorithms (OPTICS) were employed for clustering-based outlier detection. Experimental results suggest that the approach based on SAs outperforms the other approaches in both generated cluster quality and outliers' identification.

## Figures and Tables

**Algorithm 1:**
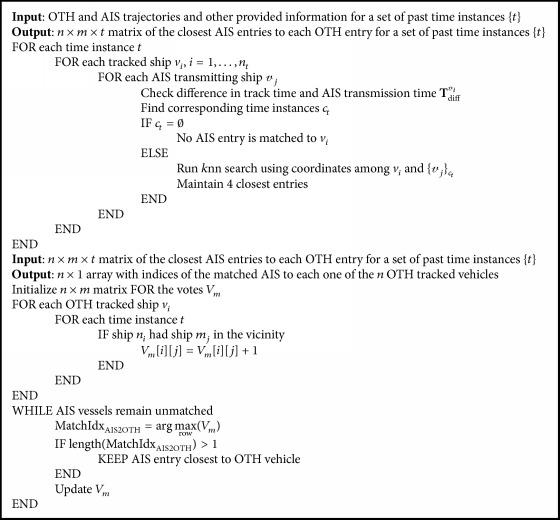
The proposed algorithm in pseudocode.

**Table 1:** Computational complexity of the different processing steps.

Processing step	Data preprocessing	Data mapping	Data clustering	OTH and AIS matching
Complexity	*O*(*n*)	*O*(*n*)	*O*(*n* · log⁡(*n*))	*O*(*n* · *m*)
